# PES/PVP Multi-Channel Mixed-Matrix Membranes with Embedded Activated Carbon for Co-Removal of Microorganisms and Extracellular DNA from Wastewater Effluent

**DOI:** 10.3390/polym18101219

**Published:** 2026-05-16

**Authors:** Jana Marx, Christian Margreiter, Verena Hettich, Christina Urban, Andreas Otto Wagner, Eva Maria Prem, Tung Pham, Martin Spruck, Jan Back

**Affiliations:** 1 Department of Environmental, Process & Energy Engineering, MCI—The Entrepreneurial School, 6020 Innsbruck, Austria; jana.marx@student.uibk.ac.at (J.M.); christian.margreiter@mci.edu (C.M.);; 2 Research Institute of Textile Chemistry and Textile Physics, Universität Innsbruck, 6850 Dornbirn, Austria; tung.pham@uibk.ac.at; 3 Institute of Microbiology, Universität Innsbruck, 6020 Innsbruck, Austria; andreas.wagner@uibk.ac.at (A.O.W.);

**Keywords:** extracellular DNA, membrane disinfection, mixed-matrix membrane filtration, advanced wastewater treatment, One Health

## Abstract

Antimicrobial resistance genes threaten the effective treatment of infectious diseases, underscoring the importance of their control in line with the EU One Health policy. Wastewater treatment plants are recognized hotspots for antimicrobial resistance. We assessed whether multi-channel mixed-matrix membranes (MCMMMs)—polyethersulfone (PES)/polyvinylpyrrolidone (PVP) ultrafiltration membranes with embedded activated carbon—can concurrently reduce microorganisms and extracellular DNA in wastewater effluent, building on prior reports of micropollutant removal. We evaluated the performance of MCMMMs in removing *Escherichia coli* and *Saccharomyces cerevisiae* as model organisms, as well as colony-forming units (CFUs) from wastewater effluent at a transmembrane pressure of 1 bar with a filtration area of 66 cm^2^ over 1 h. DNA was extracted from wastewater effluent following filtration and analyzed to assess changes in microbial community composition. MCMMMs achieved log_10_ reductions of 5.47 ± 0.42 (*Escherichia coli*), 5.99 ± 0.46 (*Saccharomyces cerevisiae*), and 2.79 ± 0.31 (wastewater CFU); reductions by pure PES/PVP membranes were comparable: higher for *Escherichia coli* and wastewater CFUs, lower for *Saccharomyces cerevisiae*. Amplicon sequencing showed altered relative abundances in wastewater effluent. Collectively, these findings demonstrate the potential of MCMMMs to simultaneously remove microorganisms, extracellular DNA, and micropollutants, highlighting their suitability for water treatment applications within the One Health framework.

## 1. Introduction

The overuse and misuse of antimicrobial agents (AAs) have led to the emergence of antimicrobial resistance genes (ARGs) in microorganisms (MOs) [1]. Many forms of resistance develop spontaneously in response to environmental stress, rendering antimicrobial treatments ineffective. Alternative treatment strategies with new antimicrobial agents must be identified; however, discovery and development are difficult and expensive. To avoid this problem in the first place, the development of new ARGs and the spread of existing ARGs must be reduced to a minimum.

Critical conditions for the development of ARGs occur at antimicrobial concentrations below the organism’s minimum inhibitory concentration (MIC). In environments, like water bodies, soil, or animals, where AAs occur but only in concentrations below the MIC, microbial growth is not inhibited, providing conditions favoring the development of ARGs [2]. For *Escherichia coli* (*E. coli*), MIC values range from 0.006 to 258 µg L^−1^ for relevant antibiotics, such as ciprofloxacin, trimethoprim, tetracycline, ampicillin, and sulfamethoxazole [3]. One environment where concentrations of pharmaceuticals below the MIC are commonly found are wastewater treatment plants (WWTPs) [4,5,6]. Ampicillin, sulfamethoxazole, ciprofloxacin, and tetracycline-resistant *E. coli* are commonly detected in wastewater effluent [7]. Hospital wastewater showed the highest percentage of resistant bacteria, such as *E. coli, Klebsiella* spp., and *Aeromonas* spp., compared to WWTP effluent, non-clinical wastewater, and surface water, mirroring the hospital antimicrobial consumption and the measured concentrations of antimicrobials [8]. In addition to low pharmaceutical concentrations, a high diversity of MOs is present in WWTPs, which leads to the second issue considered with the spread of ARGs in the environment: horizontal gene transfer. In contrast to vertical gene transfer where genetic information is transmitted to subsequent generations, some bacteria also acquire the ability for horizontal gene transfer (HGT) [9], where nucleic acids can be transferred from one bacterium to another directly via cell–cell contact or indirectly via phages or the uptake of free DNA. This underscores the need for treatment technologies that reduce the spread of antimicrobial-resistant MOs [10].

Although a reduction in biodiversity of pathogenic MOs is found during conventional wastewater treatment processes by up to 78%, a significant share of multidrug-resistant bacteria are still found in the effluent of WWTPs [11]. The release into the environment poses a threat to human health, eventually causing life-threatening diseases like cholera, typhoid fever and bacillary dysentery [12]. Effective wastewater management and treatment technologies requiring efficient removal of pathogens and their genetic material are therefore highly relevant.

Technologies to reduce pathogens in wastewater and drinking water, such as chlorination, ozonation or UV radiation [13,14,15], are broadly established [16]. Chlorine is known as the most common and effective strategy to reduce pathogens in water [17]. Additionally, advanced oxidation processes, like UV-activated persulfate, show high removal rates for antimicrobial-resistant bacteria and ARGs up to 99.90% and 76.09%, respectively [16]. However, cell lysing during chlorination and UV treatment releases genetic material including ARGs into the aqueous environment [18]. The issue of extracellular and intracellular ARGs in chlorination effluent of urban WWTPs has been demonstrated by Liu et al. [19]. Thus, even when antimicrobial-resistant organisms are removed, released DNA fragments can be taken up via HGT.

In contrast, water treatment via membrane filtration can retain MOs, ARGs, and DNA fragments. Ultrafiltration membranes with a molecular weight cut-off (MWCO) of 1 kDa can reach up to 99% removal rates for plasmids. Membranes with smaller MWCO can retain free DNA almost entirely [20], with size exclusion representing the main removal mechanism of ARGs. Adsorptive effects on the membrane material and electrostatic repulsion additionally contribute to ARG removal [21]. High pressures, membrane stability, membrane fouling and retentate disposal are key challenges [22].

Beyond membrane-based approaches, activated carbon (AC) is a mature and widely implemented option for advanced treatment of WWTP effluents, particularly effective for removing pharmaceutically active compounds [23,24,25,26]. With regard to antimicrobial resistance, granular AC has shown limited removal, with high reduction only for selected ARGs, such as beta-lactamase of *E. coli* with a 1.1 log_10_ reduction [27]. Furthermore, when AC—especially powdered AC (PAC)—is used, preventing the release of micropollutant-loaded carbon into the effluent requires downstream solid–liquid separation (e.g., sedimentation, flocculation, or filtration), increasing process complexity and costs [28]. Consequently, combined processes that couple filtration and adsorption have gained importance in quaternary wastewater treatment [29,30]. A hybrid PAC–ultrafiltration pilot process, for example, achieved log_10_ reductions of <3 and showed competitive performance for potential pathogen removal [31]. These observations motivate an integrated material solution that embeds adsorption within the membrane itself, avoiding carbon release and separate solids-removal steps.

To address these limitations, multi-channel mixed-matrix membranes (MCMMMs)—polyethersulfone (PES)/polyvinylpyrrolidone (PVP) micro-/ultrafiltration multi-channel membranes with embedded PAC—offer a single-unit operation that combines size-exclusion filtration with adsorption by the embedded PAC. MCMMM filtration makes downstream separation processes redundant, as leaching of PAC is prevented [32,33]. The successful removal of pharmaceuticals from WWTP effluent with the abovementioned MCMMM system has been demonstrated previously [32,33], with removal rates of up to 76.29 ± 4.99% for a mixture of diclofenac, carbamazepine and paracetamol [33]. In the pilot scale, this system also showed promising results for the removal of a broad range of pharmaceutically active compounds [34]. In this configuration, adsorption is the dominant mechanism for pharmaceutical removal, while filtration protects embedded PAC from competitive adsorption. Although the system was well described and characterized previously for the removal of micropollutants, the potential for an application in terms of disinfection must be still evaluated. Given the World Health Organization (WHO) prioritization of reducing ARGs [35], this study investigates whether the same combined filtration–adsorption architecture can also remove microorganisms and limit both intracellular and extracellular ARGs. By addressing pathogen removal, ARG mitigation, and micropollutant control within a single polymeric platform, MCMMMs could contribute meaningfully to wastewater treatment strategies aligned with the One Health approach. Accordingly, the aim of this study is to apply established MCMMM to wastewater effluent for microorganism and extracellular DNA removal.

## 2. Materials and Methods

**Figure 1:**
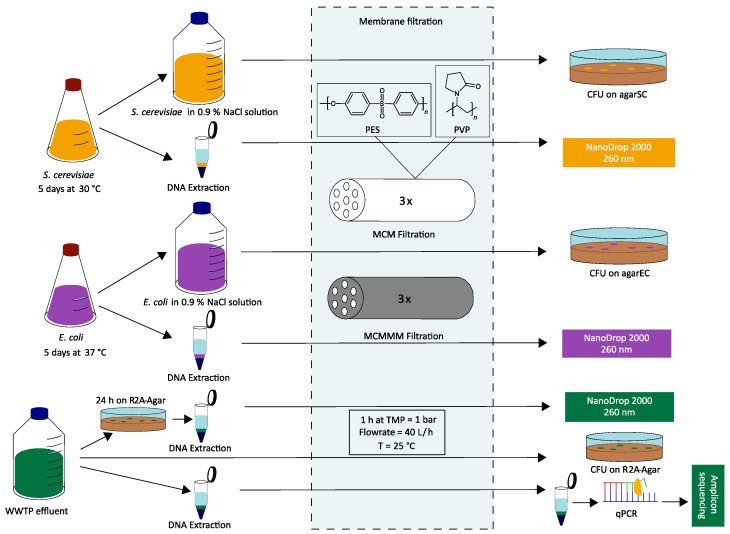
Experimental overview of the filtration of the model organisms *S. cerevisiae* and *E. coli*, as well as their genetic material after extraction. MOs were found in WWTP effluent and the extracted DNA, and the DNA extracted from cultivated organisms found on nutrient R2 agar were filtered. All filtrations were performed in triplicate both through MCMs and MCMMMs based on polyethersulfone (PES)/polyvinylpyrrolidone (PVP). Quantification of colony-forming units on nutrient agar and photometric DNA quantification via NanoDrop 2000 post-filtration were conducted. Genetic material extracted from WWTP effluent pre- and post-filtration was analyzed by applying amplicon sequencing.

The concept of MCMMMs has been established and characterized in detail in previous studies [32,33]. In short, MCMMMs are a combined filtration and adsorption process, in which PAC (Carbopal; AP supra, Donau Carbon, Frankfurt am Main, Germany; d_10_ = 6.85 µm, d_50_ = 24.09 µm, d_90_ = 71.69 µm) is embedded into a polyethersulfone (PES)/polyvinylpyrrolidone (PVP) matrix of a multi-channel membrane with seven feed-channels fabricated in a steam–dry–wet spinning process. Membranes are stored in the non-solvent (water) over 7 days after spinning to prevent PVP leaching during filtration. The multi-channel geometry resulted in an outer diameter of 5 mm and an inner channel diameter of 1 mm. A single-fiber membrane with a length of 30 cm possesses an active filtration area of 66 cm^2^. Embedding of PAC particles enhanced the BET surface area of MCM from 13.1 m^2^ g^−1^ to 145.2 m^2^ g^−1^ for MCMMMs by simultaneously increasing the surface roughness [33]. The MCMMMs applied in this study have a solid mass share of 24.0 wt% PAC. Macromolecular retention using 500 kDa dextran (75.8 ± 1.7%) indicated a pore size range of open ultrafiltration/tight microfiltration. MCMMMs are stable up to a burst pressure of 2.78 ± 0.51 bar and withstood a tensile strength of 31.58 ± 0.94 N mm^−2^ [32]. PAC incorporated well into the membrane matrix, with no PAC leaching observed during filtration. Pure PES/PVP multi-channel membranes (MCMs; without embedded PAC) and MCMMMs (with embedded PAC) are compared regarding their removal of MOs and of extracted DNA from aqueous solutions. An overview of the experimental setup is given in Figure 1.

### 2.1. Cell Cultivation

Wastewater exhibits a variable and complex composition, encompassing not only a diverse range of pollutants but also a highly heterogeneous microbial community [36]. To elucidate the removal mechanisms involved in filtration and adsorption processes using MCM and MCMMM, initial filtration experiments were conducted with defined cell cultures. For this purpose, one eukaryotic and one prokaryotic microorganism, which are well-studied and easy to cultivate, were selected as model organisms: *Saccharomyces cerevisiae* (*S. cerevisiae*) and *E. coli*. These model organisms were chosen to evaluate the filtration performance of MCMs and MCMMMs for different sizes of MOs, elucidating the extent of disinfection. However, as the two MOs are not fully representative of microbial diversity in real wastewater, filtration of WWTP effluent was additionally applied.

*S. cerevisiae* was cultivated for 24 h at 30 °C on a suitable growth agar (Carl Roth, Karlsruhe, Germany) composed of 10 g glucose, 3 g yeast extract, 15 g Kobe agar, 3 g malt extract, and 5 g soy peptone per liter of deionized water. *E. coli* was grown for 24 h at 37 °C on a selective nutrient agar (Carl Roth, Karlsruhe, Germany) containing 3 g meat extract, 15 g Kobe agar, and 5 g soy peptone per liter of deionized water. Subsequently, 3–5 colonies were used to inoculate 250 mL of growth medium for each culture. The liquid growth media had the same composition as the corresponding agars, excluding Kobe agar, and were incubated by shaking for 5 days at 30 °C and 37 °C for *S. cerevisiae* and *E. coli*, respectively.

The optical density at 660 nm (OD660) was monitored to confirm mid-logarithmic growth phase. After 5 days, the cells were centrifuged three times at 3500 rpm for 5 min, with intermediate washing steps using a 0.9% sodium chloride (NaCl) solution. The resulting cell pellets were finally resuspended in 2 L of 0.9% NaCl solution, which served as the feed solution for the filtration experiments.

In addition, WWTP effluent samples were collected after treatment using the sampling unit of the municipal wastewater treatment plant in Zirl, Tyrol, Austria. The effluent was characterized by measuring pH (7.6 ± 0.4), conductivity (815.9 ± 37.7 µS cm^−1^), chemical oxygen demand (COD = 115.4 ± 64.9 mg L^−1^), and biological oxygen demand (BOD = 6.9 ± 0.1 mg L^−1^). Samples were stored at 4 °C and processed within a maximum of 48 h prior to filtration. Rapid handling of samples was prioritized to minimize interactions between MOs and diluted organic matter and to ensure representative results.

Scanning electron microscopy (SEM, JSM-IT200, JEOL, Tokyo, Japan) was applied on the inner channel surface of MCMs and MCMMMs after sputtering with a gold coating (Sputtercoater 180auto, Cressington, Watford, UK) pre- and post-filtration with *S. cerevisiae* and *E. coli* to evaluate surface changes, and biofouling caused by cell adhesion.

### 2.2. Extraction of Genetic Material

In the present study, it is assumed that DNA extracted from the selected model organisms shows widely similar behavior as extracellular DNA (eDNA) and non-cell-associated ARGs during filtration, even though fragmentation of DNA during extraction must be considered. ARGs are generally categorized as either cell-associated or non-cell-associated. Cell-associated ARGs are primarily reduced through biomass removal, whereas non-cell-associated ARGs are mainly eliminated via adsorption, degradation, or uptake by bacterial cells [21]. It was assumed that non-cell-associated ARGs exhibit filtration behavior comparable to DNA obtained from cell extracts, as eDNA often harbors an important fraction of high-risk ARGs [37,38]. This assumption is supported by findings that free extracellular DNA detected in WWTP effluent closely resembles the intracellular bacterial composition observed after secondary treatment, as well as by the co-localization of ARGs with mobile genetic elements, indicating ARG release during chlorination, as reported by Tamai et al. [39].

Genetic material from *S. cerevisiae* was extracted following cell cultivation as described above. DNA isolation was performed using a protocol adapted from Looke et al., employing lithium acetate–SDS solution for cell lysis and ethanol for DNA precipitation [40]. DNA from *E. coli* was extracted directly from liquid cultures using a combined phenol–chloroform method for cell lysis and subsequent DNA isolation [41].

For the filtration experiments targeting genetic material from wastewater, DNA was extracted from various colonies cultivated on R2A agar (Carl Roth, Karlsruhe, Germany) using the same protocol applied for *E. coli* DNA extraction [42]. Total nucleic acid concentrations were measured using the NanoDrop representing only the fraction of DNA derived from cultivated cells present in the wastewater. DNA concentrations may be overestimated due to RNA presence. Extracellular DNA and MOs not recoverable on R2A agar were excluded from this analysis. Nevertheless, this approach was necessary to investigate DNA removal mechanisms, as the concentration of free extracellular DNA in WWTP effluent is typically very low [41,43].

### 2.3. Membrane Filtration

Cross-flow filtration experiments with cell cultures were performed separately and in triplicate using both MCM and MCMMM modules with a length of 30 cm and an effective filtration area of 66 cm^2^. Filtration was carried out at room temperature for 1 h, at a constant transmembrane pressure (TMP) of 1 bar and a feed flow rate of approximately 40 L h^−1^. Samples were collected at representative time intervals throughout the filtration process.

The primary focus of the analysis was the reduction of colony-forming units (CFUs), expressed as log_10_ reduction, defined as the common logarithm of the ratio between CFU concentrations before and after treatment. Each log10 reduction corresponds to a tenfold decrease in CFU concentration. In addition, CFU concentrations in the feed were determined before and after filtration to assess CFU reduction attributable to shear forces acting on the inner surfaces of the membrane channels. CFU enumeration was conducted using appropriate nutrient agar plates. For *S. cerevisiae* and *E. coli*, the same nutrient agars used for cell cultivation were applied, whereas WWTP effluent samples were analyzed using a universal wastewater R2A agar plate (Carl Roth, Karlsruhe, Germany), which is selective for a broad range of heterotrophic MOs commonly present in wastewater.

For filtration experiments involving feed solutions containing extracted genetic material, the feed flow rate was reduced to 120 mL h^−1^ due to the limited feed volume of 250 mL. DNA concentrations were quantified using a NanoDrop 2000 spectrophotometer (Thermo Fisher, Waltham, MA, USA), with a lower limit of quantification of 2 ng μL^−1^ and absorbance measured at 260 nm. Following extraction and resuspension in 250 mL of 0.9% NaCl solution, feed DNA concentrations ranged from 21.0 to 27.6 ng μL^−1^ for *S. cerevisiae* and from 17.8 to 28.3 ng μL^−1^ for *E. coli*. DNA isolated from WWTP effluent colonies resulted in feed concentrations between 46.7 and 48.3 ng μL^−1^.

### 2.4. Amplicon Sequencing

Microbial communities present in WWTP effluent were investigated using amplicon sequencing to obtain a more detailed understanding of the microbial environment within the treatment plant. Prior to sequencing, WWTP effluent was subjected to cross-flow filtration in triplicate using single-fiber MCM and MCMMM modules with a length of 30 cm and an active filtration area of 66 cm^2^. Genetic material was extracted from samples collected before and after filtration using the ExtractNow Sewage Water DNA/RNA kit (Minerva Biolabs, Berlin, Germany). Following centrifugation, DNA was isolated from both the supernatant and the sediment and subsequently analyzed by amplicon sequencing, as described below.

The extracted DNA from WWTP effluent of feed as well as permeate of the MCMs and MCMMMs were each sequenced in triplicate. Therefore, the V4 region [44] was targeted by applying the small subunit rRNA gene primers 515f and 806r, following the Earth Microbiome Project [45]. The NGS library was prepared according to ref. [46] and modifications applied by Wunderer et al. [47]. The combined sample pool was quality-checked and sequenced using a MiSeq System (Illumina, San Diego, CA, USA) externally (Microsynth, Balgach, Switzerland). Mothur version 1.45.2 was used to process the raw data [48]. Raw sequences were assembled into contigs and quality-filtered (removing sequences with ambiguous bases, lengths outside 290–311 bp, or homopolymers >10 bp), resulting in 212,399 sequences in total and 23,600 ± 9959 sequences per sample, and then aligned to the SILVA v138.2 database [47]. After removing redundant sequences, pre-clustering, and chimera removal using VSEARCH, taxonomic classification was performed using the Wang classifier. Non-target sequences were excluded, and OTUs were clustered at 97% similarity. Alpha diversity (Shannon index, species richness, and Pielou’s evenness) was calculated from absolute OTU abundances using the vegan package in R 4.4.1 [49,50]. Beta diversity was assessed via Bray–Curtis dissimilarity. Feed-based core microbiome analysis identified OTUs present across all sample groups with a relative abundance ≥1%, which were retained in permeate samples to monitor feed reduction efficiency. 

### 2.5. Statistical Evaluation

All experiments were performed in triplicate (sample size *n* = 3 per group). Plots were created using the ggplot2 package in R 4.4.1 [51]. ANOVA was applied to test for significant differences (*p* < 0.05) in the removal efficiencies for cells and genetic material between feed and permeate and between MCMs and MCMMMs. For the core biome of the feed, statistical analysis was conducted using all feed and permeate samples. Shapiro–Wilk tests for normality and Levene’s tests for homogeneity of variance were performed (α < 0.05). Kruskal–Wallis test followed by Dunn’s post hoc test for groupwise comparison were applied alternatively to ANOVA. For calculations of overall reduction, alpha diversity, and richness, the whole sample pool was used.

## 3. Results and Discussion

### 3.1. Filtration of MOs

**Figure 2:**
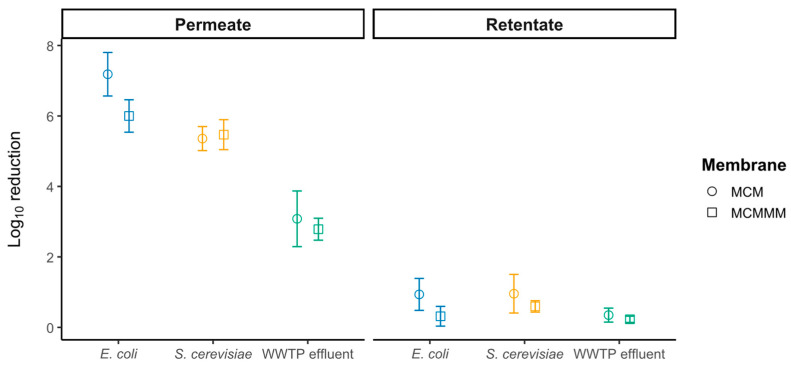
Average log_10_ reduction of *E. coli* or *S. cerevisiae* in NaCl solution, as well as from MOs in WWTP effluent in the permeate and the retentate after 60 min of cross-flow filtration with MCMs and MCMMMs (*n* = 3). Significant differences (*p* < 0.05) are seen between retentate and permeate, between MCMs and MCMMMs in permeate and retentate separately, and between *E. coli*, *S. cerevisiae* and WWTP effluent in permeate and retentate. MCMs and MCMMMs do not significantly influence the log_10_ reduction when considering permeate and retentate simultaneously (*p* > 0.05).

Cross-flow filtration of both *S. cerevisiae* and *E. coli* cells appeared to be successful for removal by MCMs and MCMMMs (Figure 2). The CFU of *S. cerevisiae* could be reduced with MCMs and MCMMMs by log_10_ 5.36 ± 0.34 and log_10_ 5.47 ± 0.43, respectively. Size exclusion is considered the main removal mechanism in this case. In comparison, *E. coli* showed an even higher retention of living cells, reaching up to log_10_ 7.18 ± 0.62 and log_10_ 5.99 ± 0.46 reduction for MCMs and MCMMMs, respectively. This could be related to the rod shape of *E. coli*, with mostly a length of 2.0–4.0 µm and a width of 0.50–1.25 µm [52]. This makes the size exclusion more variable depending on the position of the *E. coli* during filtration. Still, *S. cerevisiae* cells are on average significantly larger, with a critical diameter of around 8 µm [53]. Higher retention for *E. coli* could also be attributed to the higher permeability of MCM during *E. coli* filtration than during *S. cerevisiae* filtration. WWTP effluent filtration reached a log_10_ 3.08 ± 0.79 and a log_10_ 2.79 ± 0.31 reduction for MCM and MCMMM, respectively. 

**Figure 3:**
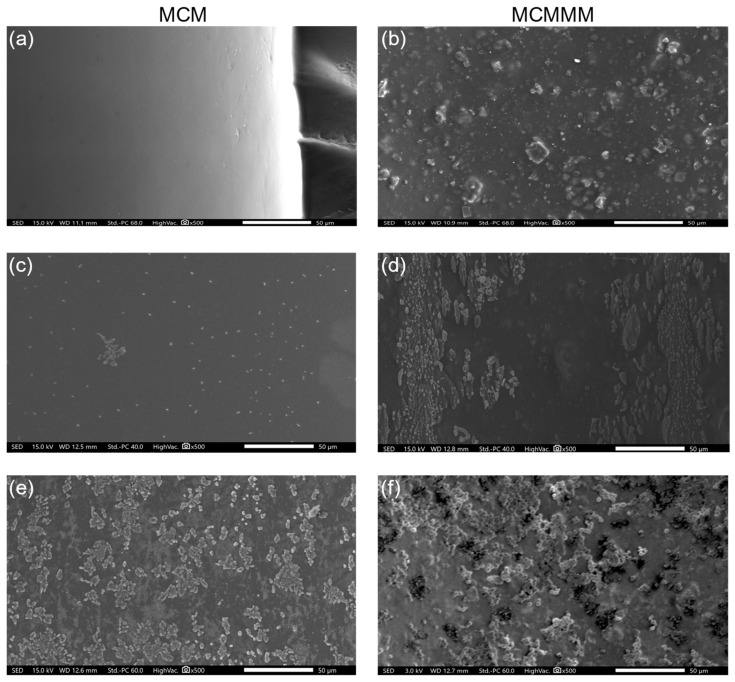
SEM images of MCM (**a**,**c**,**e**) and MCMMM (**b**,**d**,**f**) inner channel surfaces pre- (**a**,**b**) and post-filtration with *E. coli* (**c**,**d**) and *S. cerevisiae* (**e**,**f**) suspension in NaCl solution. Images are shown with a 500-fold magnification and a scale bar of 50 µm after sputtering with a gold coating.

No measurable fouling was observed over 60 min of filtration. Scanning electron microscope (SEM) imaging before and after filtration revealed cell adhesion on the inner channel surface (Figure 3). Nonetheless, the shear forces during cross-flow filtration likely inhibit cell growth overall.

The broad range of organism sizes present in WWTP effluent may reduce removal efficiency compared to that observed for the model organisms. Some MOs may be smaller than the membrane cut-off and therefore pass into the permeate. In addition, the presence of spores may contribute to reduced retention. Bacterial spores, in particular, are relatively small, typically ranging from 0.5 to 2.0 μm in size [54]. Spore formation in bacteria, such as *Clostridium* spp., is often induced by environmental stress conditions, including nutrient limitation or exposure to toxic substances [55]. Similar stress conditions may arise from shear forces during filtration, potentially promoting spore formation.

Spores exhibit high resistance to elevated temperatures and shear stress and may therefore traverse the membrane without being damaged. Under favorable growth conditions, such as those provided on agar plates, spores can become activated, germinate into vegetative cells, and subsequently form colonies. In WWTPs, spore-forming pathogens such as clostridia can survive treatment processes and disseminate into the environment, posing potential risks to human and animal health [56]. As CFU enumeration was used as the sole quantification method in this study, it was not possible to distinguish between spores and vegetative cells contributing to the observed CFU counts.

Nevertheless, the disinfection performance of MCMs and MCMMMs is comparable to that of established technologies such as UV irradiation and chlorination, which typically achieve reductions in log_10_ 2–4 [57]. As previously reported, a hybrid pilot-scale system combining PAC adsorption and ultrafiltration achieved reductions in log_10_ < 3 [31], which is within the same range as those observed for MCMMM. In contrast to conventional treatment processes, genetic material is not released through cell lysis in this case, thereby minimizing the dissemination of genomic material.

Although chlorination remains the preferred disinfection method due to its cost-effectiveness, it does not affect DNA released during cell lysis. This is evidenced by the occurrence of dissolved extracellular ARGs (eARGs) and eARGs adsorbed to particulate matter following treatment with both high and low chlorine doses [58]. In comparison, ozone treatment and TiO_2_-based photocatalysis are capable of damaging released DNA and thus reducing the spread of ARGs [59]. We compared feed CFU pre- and post-filtration to estimate shear-induced lysis (Figure 2). The observed reduction in CFUs in the retentate attributable to shear forces was log_10_ 0.23 ± 0.09 and log_10_ 0.96 ± 0.55 for MCMMMs and MCMs, respectively, indicating that cell lysis contributes only marginally to the overall CFU reduction during filtration. MCM revealed higher log_10_ reductions in the retentate compared to MCMMM, which could be caused by the lower permeability of MCMMM, causing smaller reduction in permeate flux along the membrane and therefore less change in the cross-flow velocity downstream of the membrane [60,61]. Nonetheless, the release of intracellular DNA during filtration cannot be completely excluded.

### 3.2. Filtration of Genomic Material

**Figure 4:**
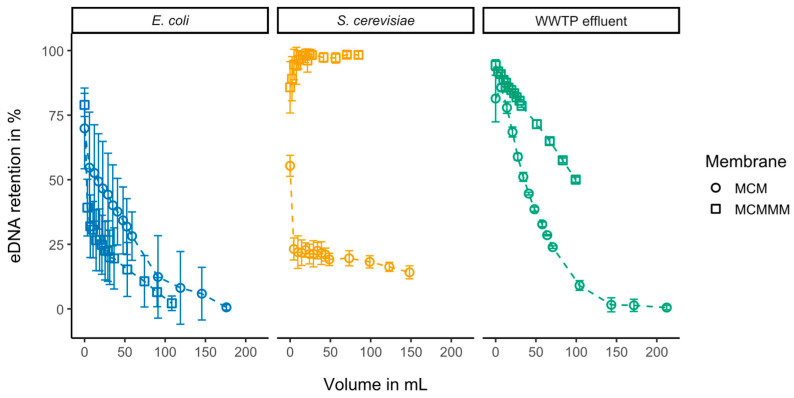
Retention of extracellular DNA (eDNA) extracted from cultivated *S. cerevisiae* cells, *E. coli* and extracted DNA of MOs cultivated from WWTP effluent after filtration with MCMs and MCMMMs over 60 min (*n* = 3). Filtered volumes are calculated based on average permeate fluxes.

Filtration of extracellular DNA (eDNA) from the two model organisms *S. cerevisiae* and *E. coli* demonstrated pronounced differences in the removal of eukaryotic versus prokaryotic genetic material (Figure 4). Eukaryotic DNA exhibited high adsorption to embedded PAC in MCMMMs with retentions of 98.33 ± 1.39% after 1 h, whereas removal with MCM, relying mainly on size exclusion, had little effect on the removal of *S. cerevisiae* DNA, resulting in retentions of 14.14 ± 2.5% after 1 h. Prokaryotic DNA from *E. coli* exhibited negligible retention in both MCMs and MCMMMs after 1 h, with removal efficiencies of 0.59 ± 1.02% and 2.17 ± 2.81%, respectively, as well as differences between feed and permeate concentrations close to the method measurement deviation. After filtering a permeate volume of 80 to 210 mL, reached over 1 h with MCMs and MCMMMs respectively, a reduction in removal efficiency is seen for *E. coli* eDNA, as well as for eDNA extracted from microorganisms found in WWTP effluent. The decline in retention is attributed to diffusion into the membrane pores at the start of filtration. Also, hydrophobic interactions with the membrane matrix could be responsible for slight removal at the start of the filtration. However, PAC adsorption is not observed for *E. coli* DNA. In WWTP effluent, adsorption exhibits sufficient reductions in DNA concentrations, due to the broad spectrum of organisms found in WWTP effluent. Inhomogeneous adsorption and reduction is seen with a downward trend throughout the filtration duration.

Adsorption and size exclusion mechanisms appear to be largely ineffective for the removal of *E. coli* genetic material. Compared to the high retentions observed for *S. cerevisiae* DNA, prokaryotic genomes are smaller and lack histone packaging. In contrast, eukaryotic DNA is primarily confined to the cell nucleus, contains a larger number of genes, and is packaged with histones [62]. Such differences in size and organization may influence removal efficiency, as molecular weight cut-off plays a role, particularly in filtration using MCM.

In addition to molecular weight cut-off, membrane surface charge significantly affects eDNA removal. MCM exhibits an acidic surface character, whereas the incorporation of PAC shifts the surface properties of MCMMMs toward an amphoteric profile, as previously described by Marx et al. [33]. Although negatively charged membranes are commonly used in wastewater treatment to minimize adsorption of natural organic matter, DNA adsorption is reduced on negatively charged surfaces compared to neutral ones [63]. Accordingly, the presence of embedded PAC in MCMMMs can be assumed to enhance DNA adsorption at the membrane surface.

Structural differences in the genetic material of the two model organisms further contribute to the observed removal behavior. *E. coli* DNA is predominantly present in circular conformations [64], whereas larger and more complex DNA structures are more readily retained during filtration due to entrapment within the porous membrane matrix. However, the substantial difference between MCMs and MCMMMs in the removal of eukaryotic DNA indicates strong adsorptive interactions between the DNA and the embedded PAC. Adsorption of DNA to PAC has already been applied with sufficient yields for DNA purification proving DNA adsorption [65]. Although both DNA and PAC are generally negatively charged and positively charged species are typically more readily adsorbed, hydrophobic interactions also play an important role in adsorption processes [66]. According to Du et al. adsorption of DNA on biochar can reach adsorption capacities of up to 296 mg g^−1^ and removal efficiencies of up to 92.7%, validating the removal through adsorption observed during MCMMM filtration [67]. Moreover, the conformational flexibility of double-stranded DNA may allow molecules to deform and pass through membrane pores [68]. Taken together, the combined effects of filtration and adsorption provide plausible explanations for the differing removal efficiencies observed for eukaryotic and prokaryotic DNA beyond size exclusion alone. This distinction is particularly important when interpreting the retention behavior of mixed DNA extracted from cultivated MOs in WWTP effluent.

Similarly to *E. coli*, extracted DNA from MOs cultivated from WWTP effluent exhibited higher removal efficiencies with MCMMMs compared to MCMs (Figure 4). When filtering WWTP effluent, competitive adsorption with dissolved organic matter (DOM) and micropollutants must be taken into account. In addition, other constituents present in wastewater can influence eDNA removal through adsorption processes. Heavy metals, for example, have been shown to enhance eDNA adsorption by inducing DNA strand unwinding and strengthening hydrogen bonding interactions with biochar [69]. Such effects may also occur in wastewater effluent, where heavy metals are typically present at low concentrations [70], potentially promoting eDNA removal via adsorption.

Conversely, membrane-based removal efficiencies of eDNA can be increased in wastewater compared to buffer solutions due to the formation of complexes between eDNA and wastewater colloids [71]. Dissolved organic matter, while acting as a major competitor for eDNA during adsorption, also contributes to colloid formation. As a result, DOM may simultaneously play a beneficial role in enhancing eDNA removal from wastewater during filtration processes [72,73]. According to Merkler et al., eDNA concentrations were higher in the effluent from the secondary clarifier compared to the influent of the WWTP, suggesting that cell lysis takes place during the treatment process [31].

**Figure 5:**
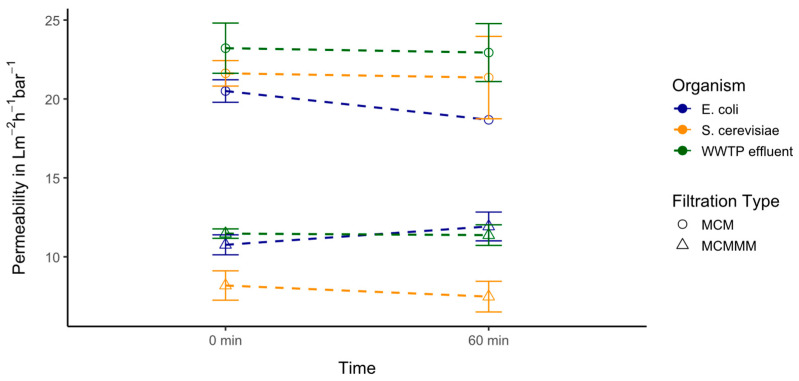
Permeability of MCMs and MCMMMs before (0 min) and after (60 min) cross-flow filtration with *E. coli*, *S. cerevisiae* and WWTP effluent. Average and standard deviation of gravimetrically determined permeate flow are shown (*n* = 3).

MCMMM permeabilities are generally lower than those of MCM (Figure 5). Most likely this is attributed to pore blocking through the embedded PAC particles [32]. Permeabilities of MCMs and MCMMMs did not significantly decline over a filtration time of 60 min, even at high MO concentrations (*p* = 0.776). Differences mainly occur due to the embedded PAC in the MCMMMs, lowering the permeate flux significantly (*p* = 1.36 × 10^−15^). This suggests that over the filtration time of 60 min, no visible fouling is taking place.

Still, fouling has to be considered in the context of biomass filtration. In particular, biofouling through growth of MOs in the form of biofilms on the surface of the membrane has a high potential for negative effects on the filtration performance. Biofilms can act as reservoirs for ARGs by protecting the cells from environmental stresses [74]. High cell density and close proximity of cells can enhance also the exchange of ARGs through HGT. However, transmission of DNA, especially plasmids, increases with higher permeate flux due to flow induced plasmid elongation [75], which is mainly relevant for the high permeability seen in MCM compared to MCMMM. In general, cross-flow filtration should reduce fouling due to shear forces occurring on the channel surface. Still, actual effects of biofouling on the MCMMM filtration have to be evaluated, but based on results for the regeneration and backwashing of MCMs and MCMMMs after micropollutant filtration, the recovery of the filtration performance and the adsorption capacity appear promising [32]. Filtration of WWTP effluent with MCMMM modules over a total of 144 h have previously shown limited effects of fouling. Chemical regeneration using an aqueous ethanol solution restored the cumulative removal of pharmaceuticals to 76.3%, suggesting sufficient desorption from PAC. However, an increase in permeate flux was observed after regeneration suggesting a change in the membrane composition through partial PVP leaching [34]. This low fouling susceptibility is attributed to the combination of chemical regeneration and shear forces occurring during cross-flow filtration and the antifouling properties observed, for example, for embedded graphene nanoplatelets in composite membranes [76].

### 3.3. Microbial Diversity Based on Amplicon Sequencing

**Figure 6:**
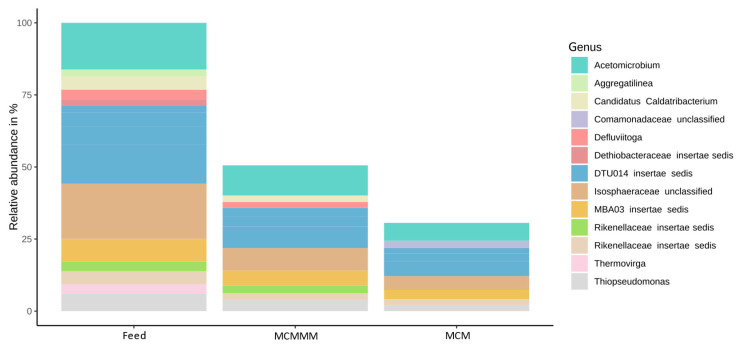
Relative abundance of the core biome in feed and permeate samples of MCM and MCMMM. Filtrations were performed in triplicate and the core biome represents all genera occurring with relative abundances ≥5% in the feed samples.

Membrane filtration altered the composition of the feed core biome significantly (Figure 6). In case of pure PES MCM filtration, the relative abundance of the feed core biome decreased to 30. 62% (*p* < 0.001), whereas the reduction through MCMMMs amounted to 50.58% (*p* = 0.010). Alpha diversity (α-D) and richness (R) appeared similar between feed (α-D = 4.58 ± 0.07, R = 534.0 ± 26.3) and permeate samples, as well as between the permeate of the MCM (α-D = 2.89 ± 1.06, R = 580.0 ± 43.6) and the MCMMMs (α-D = 3.65 ± 1.12, R = 593.0 ± 126.0), with no significant differences (*p* > 0.05). Conventional and advanced wastewater treatment steps should preferably reduce wastewater biodiversity, especially when considering ARG and antimicrobial-resistant organisms as part of the biodiversity and the species richness [77]. A similar alpha diversity and richness among all samples suggested a more or less similar reduction of all species simultaneously. However, *Dethiobacteraceae incertae sedis*, *Aggregatilinea* sp. and *Thermovirga* sp. were completely removed from the core biome with both MCM and MCMMM filtration, and *Defluvitoga* sp., *Rikenellaceae insertae sedis* sp. and *Candidatus caldatribacterium* sp. were only eliminated through MCM filtration. This difference in removal might result from the rougher surface of MCMMMs compared to MCMs due to the PAC particles found on the inner channel of the membrane, which has been previously observed by scanning electron microscopy and atomic force microscopy [33]. Rapid cell adhesion and biofilm formation are promoted by rougher surfaces of membranes [78].

In this study, ARGs were not determined, as no ARGs from the analyzed spectrum of ARGs were found in the conducted target ARG analysis from the wastewater effluent samples. Antimicrobial-resistant organisms are generally found in wastewater effluent; although a reduction is usually already seen during the conventional treatment process, the relevance of the release of pathogens and antimicrobial-resistant organisms from WWTPs is high considering the One Health approach and the potential reuse of wastewater effluent in water scarcity scenarios [79]. Influents and effluents monitored over one year in WWTPs in Italy showed reduction in the total abundance of ARGs and antimicrobial-resistant bacteria but an increase in the diversity during conventional wastewater treatment [80]. Still, it can be stated that ARGs found in municipal WWTPs highly depend on the composition of the influent of the WWTP. This composition could potentially be influenced by the discharge of wastewater from the hospital near the considered WWTP. However, according to Muoghalu et al., domestic wastewater shows highest relative ARG abundance compared to hospital wastewater, although greater ARG diversity was found in the latter [81]. Depending on pre-treatments and variation in contamination through excretion of the patients, the diversity and abundance of the determined antimicrobial-resistant organisms and ARGs might differ significantly [43]. Pathogens are in many cases already reduced during conventional wastewater treatment and MOs, which are characteristic of activated sludge, are found in larger abundance in WWTP effluent [77]. In this study, only the treatment of the effluent by advanced purification processes is considered, and no conclusion about the reduction in microorganisms during previous treatment steps can be drawn. However, the method is limited by the genera detected in WWTP effluent, and resistances appearing in single species were not determined. Some genera found in the core biome are characteristic MOs for activated sludge treatment, like *Acetomicrobium* sp. and *Comamonadaceae unclassified* sp. [82,83]. In general, membrane filtration can reduce ARGs by up to ~90% in WWTP effluent, depending on the pore size [81]. Removal of ARGs and pathogens by MCMMM filtration has to be evaluated in future work by experimental validation. Still, the presented results suggest large potential for the reduction for a wide range of microorganisms and their genetic material from wastewater effluent by filtration.

## 4. Conclusions

In the post-pandemic decade, amidst the complex interaction of humans, animals, and the environment, One Health has gained renewed importance. Building on prior pharmaceutical removal with MCMMMs (up to 76.29 ± 4.99%), this study shows that MCMMMs—PES/PVP micro-/ultrafiltration multi-channel membranes with embedded PAC—can concurrently reduce microorganisms and extracellular DNA (eDNA) from WWTP effluent. Over 60 min of cross-flow filtration, MCMMMs achieved log_10_ reductions of 5.99 ± 0.46 for *E. coli*, 5.47 ± 0.42 for *S. cerevisiae*, and 2.79 ± 0.31 for WWTP effluent CFUs. Eukaryotic DNA was strongly retained (up to 98.33%), whereas prokaryotic DNA showed limited removal under the tested conditions, indicating a major role of PAC-driven adsorption in eDNA elimination. Amplicon sequencing revealed a 50.58% reduction of the feed core biome after MCMMM filtration, with no detectable loss of hydraulic performance over 60 min.

The short filtration window limited evaluation of biofouling and performance decline during biomass filtration. *S. cerevisiae* and *E. coli* were only partially representative for the broader WWTP effluent microbiome resolved by amplicon sequencing. ARGs were not quantified, constraining conclusions to eDNA from cultivated organisms in wastewater effluent. Therefore, long-term fouling, regeneration, and retentate management as well as performance and economics at pilot/full scale should be assessed in future studies, aligned with existing pilot-scale MCMMM work on pharmaceutical removal.

These findings position MCMMMs as a single-unit operation that combines size exclusion and embedded-PAC adsorption for quaternary effluent treatment. Based on the demonstrated microorganism and eDNA removal, future work should aim to limit the spread of pathogens and resistances in the environment. Horizontal gene transfer, which mainly occurs in bacterial cells, could be reduced by optimizing the process toward prokaryotic DNA removal. Although this study did not quantify ARGs, resolving ARG dynamics remains essential. Future work should (i) quantify ARGs across seasons and operating conditions via extended qPCR/dPCR and metagenomics and (ii) elucidate adsorption mechanisms for DNA of different sizes and conformations. The successful disinfection and promising eDNA removal shown here support integrating MCMMM filtration into quaternary treatment to help mitigate pathogen dissemination and ARG propagation in WWTP effluents.

## Data Availability

The original contributions presented in this study are included in the article. Further inquiries can be directed to the corresponding author.

## Abbreviations

The following abbreviations are used in this manuscript:

AAs	Antimicrobial agents
AC	Activated carbon
ARGs	Antimicrobial resistance genes
BOD	Biological oxygen demand
CFU	Colony-forming units
COD	Chemical oxygen demand
DOM	Dissolved organic matter
*E. coli*	*Escherichia coli*
eARGs	Extracellular ARGs
eDNA	Extracellular DNA
HGT	Horizontal gene transfer
MCM	Multi-channel membrane
MCMMM	Multi-channel mixed-matrix membrane
MIC	Minimum inhibitory concentration
MO	Microorganism
MWCO	Molecular weight cut-off
NaCl	Sodium chloride
PAC	Powdered activated carbon
PES	Polyethersulfone
PVP	Polyvinylpyrrolidone
R	Richness
*S. cerevisiae*	*Saccharomyces cerevisiae*
TMP	Transmembrane pressure
WHO	World Health Organization
WWTP	Wastewater treatment plant
α-D	Alpha diversity
